# Ensemble learning of diffractive optical networks

**DOI:** 10.1038/s41377-020-00446-w

**Published:** 2021-01-11

**Authors:** Md Sadman Sakib Rahman, Jingxi Li, Deniz Mengu, Yair Rivenson, Aydogan Ozcan

**Affiliations:** 1 Electrical and Computer Engineering Department, University of California, Los Angeles, CA 90095 USA; 2 Bioengineering Department, University of California, Los Angeles, CA 90095 USA; 3 California NanoSystems Institute (CNSI), University of California, Los Angeles, CA 90095 USA

**Keywords:** Imaging and sensing, Applied optics, Optical physics

## Abstract

A plethora of research advances have emerged in the fields of optics and photonics that benefit from harnessing the power of machine learning. Specifically, there has been a revival of interest in optical computing hardware due to its potential advantages for machine learning tasks in terms of parallelization, power efficiency and computation speed. Diffractive deep neural networks (D^2^NNs) form such an optical computing framework that benefits from deep learning-based design of successive diffractive layers to all-optically process information as the input light diffracts through these passive layers. D^2^NNs have demonstrated success in various tasks, including object classification, the spectral encoding of information, optical pulse shaping and imaging. Here, we substantially improve the inference performance of diffractive optical networks using feature engineering and ensemble learning. After independently training 1252 D^2^NNs that were diversely engineered with a variety of passive input filters, we applied a pruning algorithm to select an optimized ensemble of D^2^NNs that collectively improved the image classification accuracy. Through this pruning, we numerically demonstrated that ensembles of *N* = 14 and *N* = 30 D^2^NNs achieve blind testing accuracies of 61.14 ± 0.23% and 62.13 ± 0.05%, respectively, on the classification of CIFAR-10 test images, providing an inference improvement of >16% compared to the average performance of the individual D^2^NNs within each ensemble. These results constitute the highest inference accuracies achieved to date by any diffractive optical neural network design on the same dataset and might provide a significant leap to extend the application space of diffractive optical image classification and machine vision systems.

## Diffractive networks light the way for better optical image classification

Scientists in USA have demonstrated significant improvements in the performance of diffractive optical networks, marking a major step forward for their use in optics-based computation and machine learning. There is renewed interest in optical computing hardware due to its potential advantages, including parallelization, power efficiency, and computation speed. Diffractive optical networks utilize deep learning-based design of successive diffractive layers to all-optically process information as the light is transmitted from the input to the output plane. Led by Aydogan Ozcan, a team of researchers from University of California, Los Angeles has significantly improved the statistical inference performance of diffractive optical networks using feature engineering and ensemble learning. Using a pruning algorithm, they searched through 1,252 unique diffractive networks to design ensembles of desired size that substantially improve the overall system’s all-optical image classification accuracy.

## Introduction

Recent years have witnessed the emergence of deep learning^1^, which has facilitated powerful solutions to an array of intricate problems in artificial intelligence, including image classification^2,3^, object detection^4^, natural language processing^5^, speech processing^6^, bioinformatics^7^, optical microscopy^8,9^, holography^10–12^, sensing^13^, and many more^14^. Deep learning has become particularly popular because of the recent advances in the development of advanced computing hardware and the availability of large amounts of data for training deep neural networks. Algorithms such as stochastic gradient descent and error backpropagation enable deep neural networks to learn the mapping between an input and the target output distribution by processing a large number of examples. Motivated by this major success enabled by deep learning, there has also been a revival of interest in optical computing^15–28^, which has some important and appealing features, such as (1) parallelism provided by optics/photonics systems, (2) potentially improved power efficiency through passive and/or low-loss optical interactions, and (3) minimal latency.

As a recent example of an entirely passive optical computing system, diffractive deep neural networks (D^2^NNs)^18,23,25,29–34^ have been demonstrated to perform all-optical inference and image classification through the modulation of input optical waves by successive diffractive surfaces trained by deep learning methods, e.g., stochastic gradient descent and error backpropagation. Earlier generations of these diffractive neural networks achieved >98% blind testing accuracies in the classification of handwritten digits (MNIST) encoded in the amplitude or phase channels of the input optical fields and were experimentally demonstrated using terahertz wavelengths along with 3D printing of the resulting diffractive layers/surfaces that form a physical network. In a D^2^NN fabricated with linear materials in which nonlinear optical processes including surface nonlinearities are negligible, the only form of nonlinearity in the forward optical model occurs at the opto-electronic detector plane. Without the use of any nonlinear activation function, the D^2^NN framework still exhibits depth feature as its statistical inference and generalization capabilities improve with additional diffractive layers, which was demonstrated both empirically^18,25^ and theoretically^34^. The same diffractive processing framework of D^2^NNs has also been utilized to design deterministic optical components, e.g., ultra-short pulse shaping, spectral filtering and wavelength division multiplexing^30,32^.

**Fig. 1:**
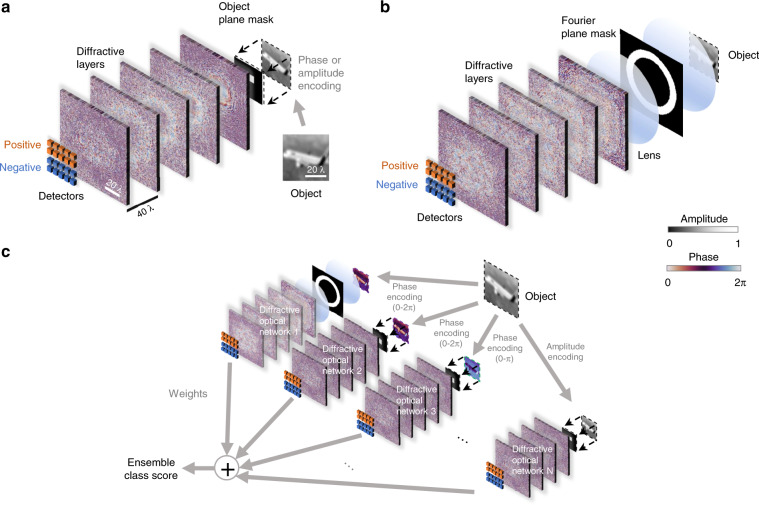
Schematic diagram of the ensemble diffractive network system. **a** Example of a D^2^NN using a feature-engineered input, where an input mask with a passive transmission window opened at a certain position is employed against the object plane. An object from the CIFAR-10 image dataset is shown as an example and is encoded in either the amplitude channel or the phase channel of the input plane of the diffractive network. **b** Same as in (**a**) but using a passive input mask placed on the Fourier plane of a 4-f system; here, a bandpass filter is shown as an example. **c** An ensemble D^2^NN system, formed by N different feature-engineered D^2^NNs, is shown where each diffractive network of the ensemble takes the form of (**a**) or (**b**). The final ensemble class score is computed through a weighted summation of the differential detector signals obtained from the individual diffractive networks. Through feature engineering and ensemble learning, we achieved blind inference accuracies of 62.13 ± 0.05%, 61.14 ± 0.23% and 60.35 ± 0.39% on the CIFAR-10 test image dataset using *N* = 30, *N* = 14 and *N* = 12 D^2^NNs, respectively. The standard deviations are calculated through 3 repeats using the same hyperparameters

To further improve the inference capabilities of optical computing hardware, coupling diffractive optical systems with jointly trained electronic neural networks that form opto-electronic hybrid systems has also been reported^19,25,29^, where the front end is optical/diffractive and the back end is all-electronic. Despite all this progress, there is still much room for further improvements in the diffractive processing of optical information. Here, we demonstrate major advances in the optical inference and generalization capabilities of the D^2^NN framework by feature engineering and ensemble learning over multiple independently trained diffractive neural networks, where we exploit the parallel processing of optical information. To create this advancement, we first focus on diversifying the base D^2^NN models by manipulating their training inputs by means of spatial feature engineering. In this approach, the input fields are filtered in either the object space or the Fourier space by introducing an assortment of curated passive filters before the diffractive networks (see Fig. 1). Following the individual training of 1252 unique D^2^NNs with various features, we used an iterative pruning strategy to obtain ensembles of D^2^NNs that work in parallel to improve the final classification accuracy by combining the decisions of the individual diffractive classifiers. Based on this feature engineering and iterative pruning strategy, we numerically achieved blind testing accuracies of 61.14 ± 0.23% and 62.13 ± 0.05% (referring to the mean ± standard deviation, which was calculated using three independent runs) on the classification of CIFAR-10^35^ test images with ensemble sizes of *N* = 14 and *N* = 30, respectively. Stated differently, 14 D^2^NNs (30 D^2^NNs) selected through this pruning approach work in parallel to collectively reach an optical inference accuracy of 61.14 ± 0.23% (62.13 ± 0.05%) on the CIFAR-10 test images, which provides an improvement of >16% over the average classification accuracy of the individual D^2^NNs within each ensemble, demonstrating the ‘*wisdom of the crowd’.* This image classification performance is the highest achieved to date by any diffractive optical network design applied on the same dataset. We believe that this substantially improved inference and generalization performance provided by feature engineering and ensemble learning of D^2^NNs marks a major step in opening up new avenues for optics-based computation, machine learning and machine vision-related systems, benefiting from the parallelism of optical systems.

## Results

Ensemble learning refers to improving the inference capability of a system by training multiple models instead of a single model and combining the predictions of the constituent models (known as base models, base learners or inducers). It is also possible to learn how to combine the decisions of the base learners, which is known as meta-learning^36^ (learning from learners). Ensemble learning is beneficial for several reasons^37^; if the size of the training data is small, the base learners are prone to overfitting and, as a result, suffer from poor generalizability to unseen data. Combining multiple base learners helps to ameliorate this problem. In addition, by combining different models, the hypothesis space can be extended, and the probability of getting stuck in a local minimum is reduced. An important aspect to consider when generating ensembles is the diversity of the learned base models^37^. The learned models should be diverse enough to ensure that different models learn from different attributes of the data, such that through their ‘collective wisdom’, the ensemble of these models can eliminate the implicit variance of the constituent models and substantially improve the collective inference performance. One approach to enrich the diversity of the base models is to manipulate the training data used to train different classifiers, making them learn different features of the input space in each trained model. In addition to the training of these unique and independent classifiers, pruning methods that aim at finding small ensembles while also achieving competitive inference performance are also very important^37^.

Based on these considerations, Fig. 1a, b depict the two types of D^2^NNs^29^ (base learners) selected to constitute our ensemble diffractive system. The difference between these two types lies in the placement of the input mask (passive) used to filter out different spatial features of the object field to variegate the information fed to the base D^2^NN classifiers. In the structure of Fig. 1a, the input filter is placed on the object plane, whereas the structure of Fig. 1b uses an input filter on the Fourier plane of a 4-f system placed before the D^2^NN. Further heterogeneity is introduced by diversifying the input filter profiles for both types of D^2^NNs depicted in Fig. 1a, b (see Supplementary Table S1). For example, input filters with transmissive windows of different shapes (rectangular, Gaussian, Hamming, or Hanning windows) and in different locations are used at the object plane. The input filters used at the Fourier plane also vary in terms of their pass/stop bands (see the “Materials and methods” section for more details). In designing the object plane filters, we used windows of various shapes and sizes and in various locations to help the individual D^2^NNs independently learn the object features at different spatial positions and windows of the input plane. Similar considerations were also made during the design of the Fourier plane filters. Although a filtering operation at the Fourier plane can be represented by an equivalent convolution on the object plane, the two types of input filters serve different purposes. The spatial domain filters provide attention (similar to the attention mechanism used in deep learning^38^) to spatial features and regions of interest at the input plane, while the Fourier plane filters provide different engineered point spread functions and convolution operations that are uniformly applied over the entire sample field of view; in this sense, these two sets of filters complement each other in the desired inference task.

To further improve the diversity of the base models, the input object information is encoded into either the phase channel with four different dynamic ranges or the amplitude channel of the illumination field. Using all of these different hyperparameter choices and their combinations, 1252 unique D^2^NN classifiers were trained to form the initial network pool. In total, 340 of these networks had the input object information encoded in the amplitude channel, while 912 of them had phase-encoded inputs; 276 of the amplitude-encoded D^2^NNs had an input filter located on the object plane, and 64 had an input filter located on the Fourier plane; 656 of the phase-encoded-input networks had a filter on the object plane, and 256 had a filter on the Fourier plane. For these 1252 unique D^2^NN classifiers, each diffractive neural network subsequently acts on the filtered version of the input image, and therefore, the trained diffractive layers of each base D^2^NN directly act on the space domain information (not on the frequency or Fourier domain).

The preparation of this initial set of 1252 unique D^2^NNs was followed by iterative pruning, with the aim of obtaining ensembles of significantly reduced size, i.e., with a much smaller number of D^2^NNs (base models) in the ensemble. Ensemble pruning was performed by assigning weights to each class score provided by the individual D^2^NN classifiers and defining the ensemble class score as a weighted sum of the individual class scores. At each iteration of ensemble pruning, the weights were optimized through gradient descent and error backpropagation method to minimize the softmax-cross-entropy (SCE) loss between the predicted ensemble class scores and their one-hot labelled ground truth, and the set of weights providing the highest accuracy were chosen (see the “Materials and methods” section). Then, the ‘significance’ of the individual D^2^NNs in a given state of the ensemble was quantified and ranked by the absolute summation (i.e., the L1 norm) of their weights, based on which a certain fraction of the networks was then eliminated from the ensemble due to their relatively minor contributions. In addition to this greedy search, periodic *random* elimination of the individual classifiers from the ensemble was also used in the pruning process to expand the solution space (see the “Materials and methods” section for details).

**Fig. 2:**
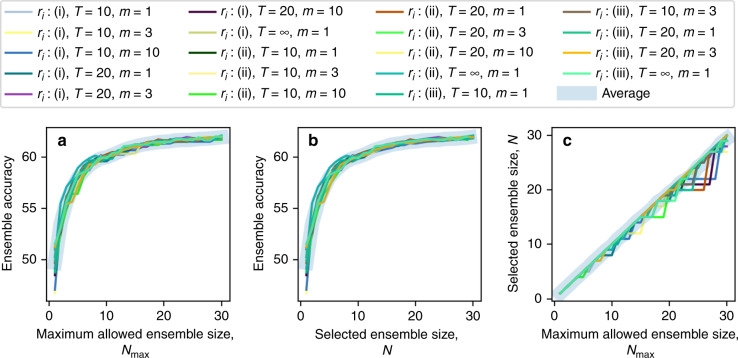
Inference accuracy of the D^2^NN ensembles as a function of *N*_max_ and N. **a** Variation in the blind testing accuracy as a function of the maximum allowed ensemble size (*N*_max_) during the pruning; **b** Variation in the blind testing accuracy as a function of the selected ensemble size (*N*); **c** Relationship between *N*_max_ and *N*. The symbols in the legend denote different pruning hyperparameters used in our ensemble selection process; also see Fig. 4 and Table 1

Based on this pruning process, the iterative search algorithm resulted in a sequence of D^2^NN ensembles with gradually decreasing sizes. To select the final ensemble with a desired size (i.e., the number of unique networks), we set a maximum limit on the ensemble size (referred to as the ‘maximum allowed ensemble size’, i.e., *N*_max_) and searched for the D^2^NN ensemble that achieves the best performance in terms of inference accuracy on the validation dataset (i.e., the test dataset was never used during the pruning phase). As we followed this procedure for different values of the pruning hyperparameters, D^2^NN ensembles with different sizes and blind testing accuracies were created; we repeated our search three times for each set of hyperparameters, which helped us quantify the mean and standard deviation of the inference accuracy for the resulting D^2^NN ensembles. We repeated the pruning process three times for each combination of hyperparameters and reported the mean and standard deviation over these repeats in the form of mean ± standard deviation. Based on these analyses, Fig. 2a reveals that as the maximum allowed ensemble size (*N*_max_) increases, the blind testing accuracies increase; Fig. 2b shows a similar trend reporting the blind testing accuracies as a function of *N*, i.e., the number of D^2^NNs in the selected ensemble. Figure 2c further reports the relationship between *N* and *N*_max_ during the pruning process, which indicates that on average, these two quantities vary linearly (with a slope of ~1).

**Fig. 3:**
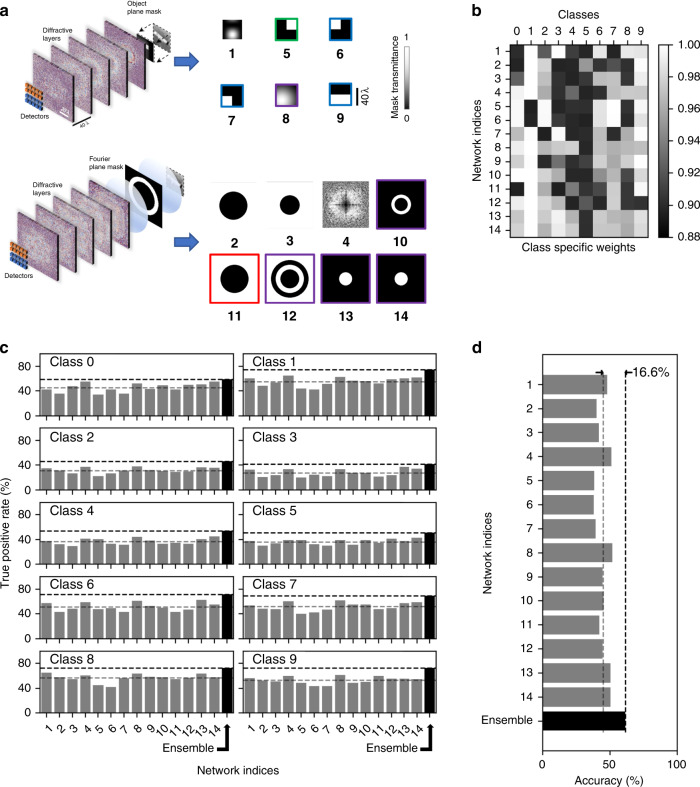
An ensemble of *N* = 14 D^2^NNs achieves a blind classification accuracy of 61.21% on the CIFAR-10 test dataset. **a** Input filters/masks used before each of the D^2^NNs that form the ensemble. For D^2^NNs 1 and 5–9, the input filters are on the object plane. For the remaining D^2^NNs (i.e., D^2^NNs 2–4 and 10–14), the input filters are on the Fourier plane. The input filters corresponding to the networks with phase-encoded inputs are enclosed within a border/frame (D^2^NNs 5–14), while the inputs of D^2^NNs 1–4 are amplitude-encoded. The dynamic range of the input phase encoding is represented by the border colour; red: 0–π/2, green: 0–π, blue: 0–3π/2, purple: 0–2π. **b** Class-specific weights for each D^2^NN of the ensemble. If one ignores these class-specific weights and replaces them with all ones, the blind inference accuracy slightly decreases to 61.08%, from 61.21%. **c** True positive rates of the individual diffractive networks compared against their ensemble for different classes. **d** Test accuracy of the individual networks compared against their ensemble. The dotted lines show the classification performance improvement (~16.6%) achieved by the diffractive ensemble over the mean performance of the individual D^2^NNs. Three repeats with the same hyperparameters resulted in a blind classification accuracy of 61.14 ± 0.23%, where 61.21% represents the median, as detailed in this figure

While the results reported in Fig. 2a, b demonstrate the significant gains achieved through the ensemble learning of diffractive networks, they also highlight a diminishing return on the blind inference accuracy of the ensemble with an increasing number of D^2^NNs selected. For example, with ensemble sizes of *N* = 14 and *N* = 30 D^2^NNs, we achieved blind inference and image classification accuracies of 61.14 ± 0.23% and 62.13 ± 0.05%, respectively, on the CIFAR-10 test dataset. Increasing the ensemble size to, e.g., *N* = 77 D^2^NNs, resulted in a classification accuracy of 62.56% on the same test dataset. Because of this diminishing return achieved by larger ensemble sizes, we further focused on the case of *N*_max_ = 14 to better explore this optimal point: Table 1 reports the blind testing accuracies (means ± standard deviations) achieved for different pruning hyperparameters for a maximum allowable ensemble size of 14. These results summarized in Table 1 reveal that, although not intuitive, the periodic random elimination of diffractive models during the pruning process results in better classification accuracies than pruning with no random model elimination; see the columns in Table 1 with *T* = ∞, where *T* refers to the interval between periodic random elimination of D^2^NN models. In Table 1, the best average blind testing accuracy (61.14 ± 0.23%) that was achieved for *N*_max_ = 14 is highlighted with a green box. For three individual repeats of the pruning process using the same hyperparameters, the classification accuracies achieved by the resulting 14 D^2^NNs were 60.88, 61.33 and 61.21%. Figure 3 further presents a detailed analysis of the latter *N* = 14 ensemble that achieved a blind testing accuracy of 61.21%, which is the median for the 3 repeats. Six of the selected base D^2^NN classifiers have input filters on the object plane, while the remaining eight have input filters on the Fourier plane (Fig. 3a). Figure 3b shows the magnitudes of the class-specific weights optimized for the base classifiers of this *N* = 14 ensemble. Even if these optimized weights are ignored and made all to be equal to 1, the same diffractive ensemble of 14 D^2^NNs achieves a similar inference accuracy of 61.08%, a small reduction from 61.21%.

**Table 1:**
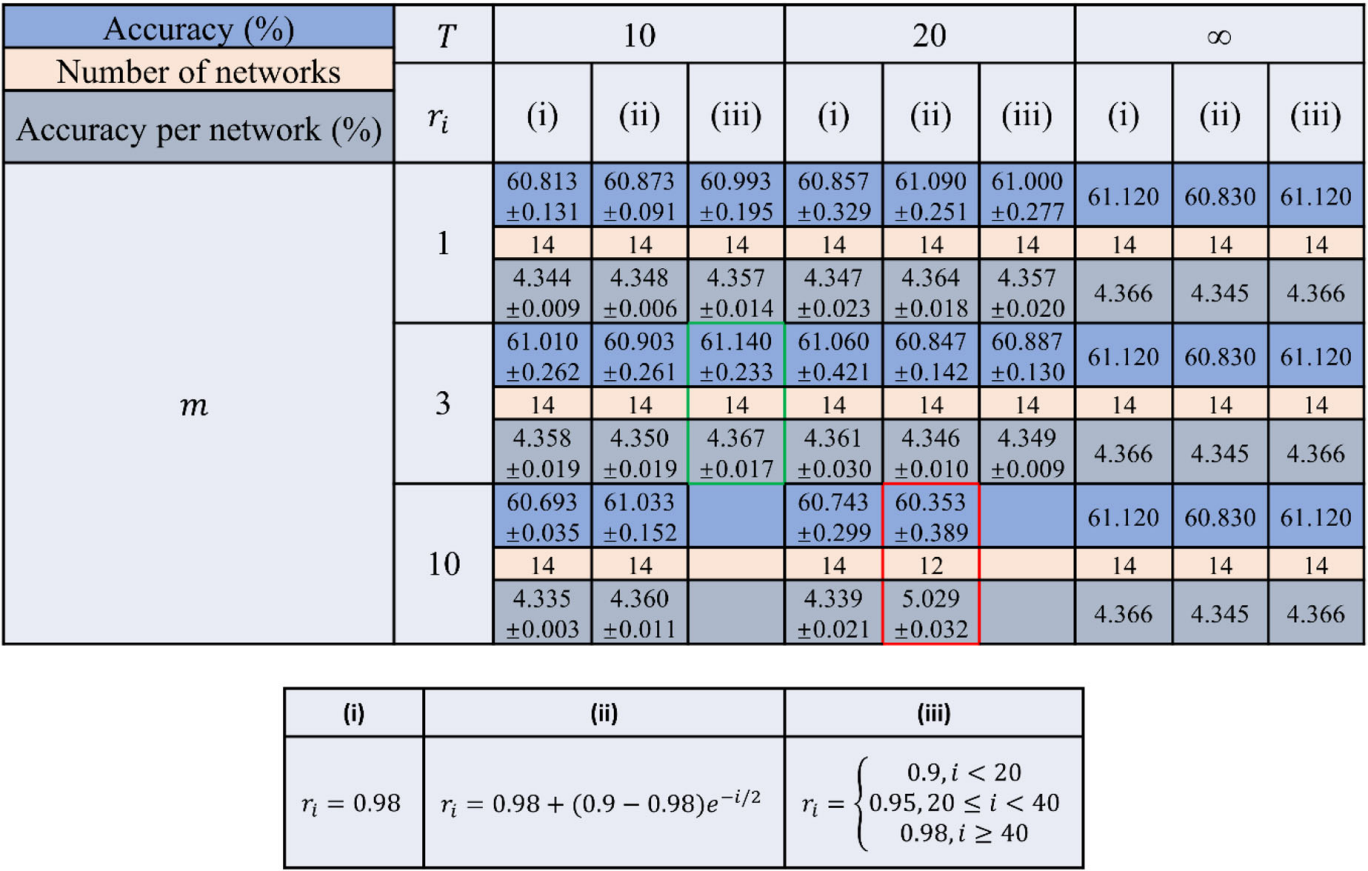
Comparison of the blind testing accuracy results achieved under different pruning hyperparameters, with a maximum allowed ensemble size of *N*_max_ = 14 (see Fig. 4).

For the reported classification accuracies, the means and standard deviations are from the three independent repeats of the pruning process using the same hyperparameters. The lower table describes the schemes used for *r*_*i*_ denoted by (i), (ii) and (iii). The green box highlights the D2NN ensemble achieving the best average blind testing accuracy (*N* = 14), and the red box highlights the D2NN ensemble achieving the best average blind testing accuracy per network (*N* = 12)

In addition, Fig. 3c shows the true positive rates for each class, corresponding to the individual members of *N* = 14 D^2^NNs as well as the ensemble. The improvements in the true positive rates of the ensemble over the mean performance of the individual classifiers for different data classes lie between 13.47% (for class 0) and 19.98% (for class 6). Figure 3d further presents a comparison of the classification accuracies of the individual diffractive classifiers compared against their ensemble. Through these comparative analyses reported in Fig. 3c, d, it is evident that the performance of the ensemble is significantly better than any individual D^2^NN in the ensemble, demonstrating the ‘wisdom of the crowd’ achieved through our pruning process.

In Table 1, we also report another metric, i.e., ‘the accuracy per network’, which is the average accuracy divided by the number of networks in the ensemble, to reveal the performance efficiency of the ensembles that achieve at least a 60% average blind testing accuracy for the CIFAR-10 test dataset. The best performance achieved in Table 1 based on this metric is highlighted with a red box: *N* = 12 unique D^2^NNs selected by the pruning process with *N*_max_ = 14 achieved a blind testing accuracy of 60.35 ± 0.39%, where the accuracy values for the individual 3 repeats were 60.77, 60.00 and 60.29%. Details of the latter ensemble with a blind testing accuracy of 60.29%, which is the median for the 3 repeats, can be found in Supplementary Fig. S1, revealing the selected input filters and the class-specific weights of the 12 D^2^NNs in this ensemble.

Our results reveal that encoding the input object information in the amplitude channel of some of the base D^2^NNs and in the phase channel of the other D^2^NNs helps to diversify the ensemble. Supplementary Table S2 further confirms this conclusion by reporting the blind testing accuracies achieved when the initial ensemble consists of *only* the 912 D^2^NNs whose input is encoded in the phase channel. A direct comparison of Table 1 and Supplementary Table S2 reveals that including both types of input encoding (phase and amplitude) within the ensemble helps improve the inference accuracy. Using only phase encoding for the input of D^2^NNs, the best average blind testing accuracy achieved using *N*_max_ = 14 was 60.74 ± 0.17% with an ensemble of *N* = 14 D^2^NNs. A detailed description of the median of these D^2^NN ensembles with a classification test accuracy of 60.65% is provided in Supplementary Fig. S2. Supplementary Fig. S3 shows the details of another phase-only input encoding ensemble with *N* = 12 D^2^NNs, achieving a blind testing accuracy of 60.43%.

Furthermore, it is noteworthy that the top 10 D^2^NNs in terms of their individual blind testing accuracies from the initial pool of 1252 networks were not selected in any of the D^2^NN ensembles of Fig. 3 and Supplementary Figs. S1, S2 and S3. This finding corroborates our conjecture that the individual performance of a base model might not be indicative of its performance within an ensemble. In fact, several of the base D^2^NNs selected in the ensembles of Fig. 3 and Supplementary Figs. S1, S2 and S3 had blind testing accuracies <40%, whereas the blind testing accuracies of the best models (not chosen in any of the ensembles) were >50%.

Thus far, the pruning strategy that we have investigated is based on assigning weights to each differential class score of the individual D^2^NNs. Based on a differential detection scheme^29^, these class scores are computed through the normalized difference of the signals from the class detector pairs. To further explore whether this weight assignment can be improved, we also considered a more general case, where the trainable weights are assigned not only to the class scores but also to each of the detectors, representing a broader solution space compared to differential balanced detection^29^. We optimize this augmented set of weights in two different schemes: (1) the detector signal weights are optimized simultaneously with the class score weights in each iteration of the pruning process, and (2) the detector signal weights and the class score weights are alternatively optimized in different iterations (see the “Materials and methods” section for details). The results of these alternative pruning strategies are shown in Supplementary Tables S3 and S4. With *N*_max_ = 14, the best testing accuracy reported using optimization scheme (1) was 61.02%; when using optimization scheme (2), we achieved a blind test accuracy of 61.35%. Compared to the previous classification accuracy (61.14%) achieved using only the weights assigned to class scores, these new results present a very similar performance. This comparative analysis further confirms our previous observation that although the weights are vital for ensemble pruning, their ultimate effect on the inference accuracy is not substantial.

## Discussion

Although forming an ensemble of separately trained D^2^NNs ensues a major improvement in the classification and generalization performance of diffractive networks, further improvements could reduce the performance gap with respect to state-of-the-art electronic neural networks. The classification accuracies of widely known all-electronic classifiers on the greyscale CIFAR-10 test image dataset can be summarized as follows^29^: 37.13% for support vector machine (SVM)^39^, 66.43% for LeNet^40^, 72.64% for AlexNet^2^, and 87.54% for ResNet^3^. While the blind testing accuracy for an ensemble of *N* = 30 unique diffractive optical networks (62.13 ± 0.05%) comes close to the performance of LeNet, which was the first demonstration of a convolutional neural network (CNN), there is still a large performance gap with respect to the state-of-the-art CNNs, and this fact suggests that there might be more room for improvement, especially through a wider span of input feature engineering within larger pools of D^2^NNs, forming a much richer and more diverse initial condition for iterative pruning.

The presented improvement in the classification performance of D^2^NNs obtained with feature engineering and ensemble learning is not cost-free. Due to the multiple optical paths that are part of this framework, the number of diffractive layers and the opto-electronic detectors to be fabricated and used increases in proportion to the number of networks (N) used in the final ensemble, which results in an increased complexity for the optical network setup. The required training time also increases significantly because of the need for a large number of individual networks in the initial pool, which was 1252 individual D^2^NNs in our case. However, this training process is a one-time effort, and the inference time or latency remains the same by virtue of the parallel processing capability of the diffractive optical system; stated differently, the information processing occurs through diffraction of light within each D^2^NN of the ensemble, and because all of the individual diffractive networks of an ensemble are passive devices that work in parallel, we do not expect a slowdown in the inference speed. In addition, the detection circuitry complexity of the diffractive optics-based solutions is still minimal compared to its electronic counterparts, and the hardware complexity of D^2^NN ensembles can be reduced even further by using an additive sum of the individual class scores instead of the weighted sum at the cost of a very small reduction in the inference accuracy. For example, for the ensemble of D^2^NNs depicted in Fig. 3, if a simple additive sum of the individual class scores is used instead of the optimized class-specific weights, the blind classification accuracy reduces only slightly from 61.21% to 61.08%. This finding suggests that a further reduction in the hardware complexity is attainable with a very small reduction in the inference accuracy by discarding the specific weights of the class scores. However, these weights still play a very significant role in the pruning process, as they help in our selection of the diffractive models to be retained in each iteration during ensemble pruning by measuring/quantifying the significance of the individual networks in an ensemble (see the “Materials and methods” section). Some of the drawbacks associated with the relatively increased size and complexity of optical hardware should also become less restrictive since advances in integrated photonics and fabrication technologies have led to continuous miniaturization of opto-electronic devices^41^. The physical dimensions of an individual D^2^NN model with a fixed number of diffractive layers are dictated by the illumination wavelength. For example, the longitudinal dimension of the D^2^NN designs used in our models is ~240 λ, which refers to the distance between the input and the output planes, and the lateral dimension is ~100 λ, which refers to the width of each diffractive layer. Using state-of-the-art fabrication technologies, it is possible to create diffractive structures with a feature size of a few hundred nanometres^42,43^, potentially extending the application of diffractive systems to, e.g., the visible spectrum. The realization of D^2^NNs in the visible spectrum would also significantly reduce the overall size of the ensemble. In addition to these 3D nanofabrication technologies based on multiphoton polymerization, multilayer photolithographic methods^44^ could also be used for the fabrication of D^2^NN systems. For the same purpose, nanoimprint lithography and roll-to-roll patterning techniques^45,46^ might be less expensive alternatives to some of these relatively costly fabrication techniques. Such miniaturized D^2^NNs operating at visible wavelengths would also present 3D alignment challenges, requiring high-resolution structuring of free-space diffractive layers, which need to be precisely aligned with each other. Recent work on the design of misalignment-resilient^31^ D^2^NN models could be useful for practical implementations of such diffractive systems operating at visible wavelengths. Furthermore, while the miniaturization of D^2^NN systems with the currently available large-area nanofabrication methods is feasible to support an ensemble of diffractive networks that operate at visible wavelengths, high-throughput fabrication and integration of miniaturized optical components such as filters and lenses might be challenging due to the relative bulkiness of such optical components. However, the recently emerging research in meta-surface-based flat optics^47,48^ has enabled significant miniaturization of traditionally bulky optical components, and this research could be further utilized for practical realizations of miniaturized D^2^NN ensembles.

In addition to the issues of hardware complexity and size, to maintain a desired signal-to-noise (SNR) ratio at the output detectors, the optical input (illumination) power of the system needs to be increased in proportion to the ensemble size. However, due to the availability of various high-power laser sources, this higher demand for illumination power of the system should not be a significant obstacle for its operation. While the use of high-power lasers might not offer a cost-effective solution, all-optical object detection and classification applications that require extremely fast inference on the spot (e.g., for threat detection) might still justify their use. In addition, since D^2^NNs are inherently passive, the availability of low-loss materials for the fabrication of diffractive layers might lead to power-efficient diffractive networks, partially offsetting the high-power illumination requirement. Furthermore, given that broadband diffractive networks have already been reported to process pulsed optical inputs^30,32,33^, the utilization of pulsed lasers, such as those that are widely used in telecommunications and microscopy applications, might help to provide sufficient SNR at each detector plane of the ensemble. Another potential solution to reduce the input power requirement could be to time-gate the illumination signals to different diffractive networks at the cost of some increase in the inference time by illuminating each individual D^2^NN of the ensemble sequentially, i.e., one by one.

The passive nature of a physically fabricated D^2^NN model, while an advantage in terms of power requirements, is also a disadvantage, as it creates limitations for dynamically changing datasets. Incorporating dynamic spatial light modulators (SLMs) to implement the diffractive layers would augment the D^2^NN framework to become reconfigurable at the cost of additional hardware complexity and power. Furthermore, diffractive networks have been shown to benefit from transfer learning, where the performance of an already fabricated D^2^NN can be improved by inserting new additional diffractive layers or replacing some of the existing diffractive layers with newly trained layers^18,32^ benefiting from the modularity of the D^2^NN design.

Another partial limitation of the proposed approach is the computation time that is needed for the training of the initial diffractive ensemble. In this paper, we trained a total of 1252 D^2^NNs, which resulted in a relatively large computational burden and a long training time. However, this is a one-time effort, and a significant reduction in the training time might be possible through further optimization of the numerical implementation of our optical forward models. Furthermore, since our investigation of the optimized ensembles after the pruning stage revealed that many types of filters were rarely represented/selected in the final ensembles (see Supplementary Table S1), there is also the possibility to significantly reduce the total number of diffractive networks to be trained as part of the initial ensemble.

Finally, the diffractive networks reported in this work utilize coherent illumination and operate at a single illumination wavelength. Recent studies have reported diffractive networks that can process a continuum of wavelengths^30,32,33^, which lends itself to the possibility of multiplexing the object information at different wavelength channels of the illumination. The inference accuracy of an ensemble diffractive model might benefit from this wavelength diversity by utilizing diffractive networks that process specific colour channels (e.g., red, green and blue), either jointly or individually. These are promising research directions for future D^2^NN ensemble designs that might further enhance their blind inference performance.

In summary, we significantly improved the statistical inference and generalization performance of D^2^NNs using feature engineering and ensemble learning. We independently trained 1252 unique D^2^NNs that were diversely engineered with various passive input filters. Using a pruning algorithm, we searched through these 1252 D^2^NNs to select an ensemble that collectively improves the image classification accuracy of the optical network. Our results revealed that ensembles of *N* = 14 and *N* = 30 D^2^NNs achieve blind testing accuracies of 61.14 ± 0.23% and 62.13 ± 0.05%, respectively, on the classification of CIFAR-10 test images, which constitute the highest inference accuracies achieved to date by any diffractive optical neural network design applied to this dataset. The versatility of the D^2^NN framework stems from its applicability to different parts of the electromagnetic spectrum and the availability of miscellaneous fabrication techniques such as 3D printing and lithography. Together with further advances in the miniaturization and fabrication of optical systems, the presented results and the underlying platform might be utilized in a variety of applications, e.g., ultrafast object classification, diffraction-based optical computing hardware, and computational imaging tasks.

## Materials and methods

### Implementation of D^2^NNs

As the basic building block of our diffractive ensemble, all the individual D^2^NN base classifiers presented in this paper consist of five successive diffractive layers, which modulate the phase of the incidence optical field and are connected to each other by free-space propagation in air. The propagation model we used was formulated based on the Rayleigh-Sommerfeld diffraction equation^18,25^, assuming that each diffractive feature (or ‘neuron’) on the diffractive layers serves as a source of modulated secondary waves, which jointly form the propagated wave field. The presented results and analyses of this manuscript are broadly applicable to any part of the electromagnetic spectrum as long as the diffractive features and the physical dimensions are accordingly scaled with respect to the wavelength of light. Using a coherent illumination wavelength of λ, for all the diffractive network designs, the size of each neuron and the axial distance between two successive diffractive layers were set to be ~0.5 λ and 40 λ, respectively, which guarantees an adequate diffraction cone for each neuron to optically communicate with all the neurons of the consecutive layer and enables the diffractive optical network to be ‘fully connected’. Each photodetector at the output plane of a D^2^NN is assumed to be a square of width 6.4 λ. Since we employed a differential detection scheme here^29^, the detectors were divided into two groups, namely, positive detectors and negative detectors, and were collectively used to compute the differential class scores for network *k*, i.e., *Z*_*ck*_, through the following equation:

1 $$z_{ck} = \frac{{z_{ck}^ + - z_{ck}^ - }}{{z_{ck}^ + + z_{ck}^ - }}$$

where $z_{ck}^ +$ and $z_{ck}^ -$ denote the optical signals from the positive and negative detectors for class *c*, respectively. Since the dataset used in this paper, i.e., the CIFAR-10 image dataset, has 10 classes, and a pair of positive and negative detectors constitutes the score for each class, therefore, there are a total of 20 detectors at the detector/output plane of a single D^2^NN. An empirical factor of *K* = 0.1, also called the ‘temperature’ coefficient in the machine learning literature^49^, was a non-trainable hyperparameter utilized to achieve more efficient convergence during the training phase by dividing Eq. 1 by *K*. In addition, the input object was encoded either in the amplitude or in the phase channel of the input illumination, which is assumed to be a uniform plane wave generated by a coherent source. The phase encoding of the input objects took values from either of the following four intervals: 0–0.5π, 0–π, 0–1.5π or 0–2π.

### Feature engineering of diffractive networks

We used two types of feature-engineered diffractive network architectures: one diffractive architecture employed an input filter placed on/against the object plane that filters the spatial signals directly, while the other architecture used an input filter placed on the Fourier plane of a 4-f system to filter certain spatial frequency components of the object. Unless the filters are specifically mentioned to be trainable, these input filter designs were pre-defined, keeping the transmittance of their pixels constant during the training of the diffractive networks (see Supplementary Table S1 for examples). Each feature-engineered diffractive network subsequently acts on the filtered input image, directly processing the input information on the spatial domain, *not* the frequency or Fourier domain.

The object plane filters are designed to be the same size as the object, containing transmissive patterns, the amplitude distribution of which takes one of the following forms: (1) 2D Gaussian functions defined with variable shapes and centre positions; (2) multiple superposed 2D Gaussian functions defined with variable centre positions; (3) 2D Hamming/Hanning functions defined with variable centre positions; (4) square windows of different sizes at variable centre positions; (5) multiple square windows at variable centre positions; (6) patch-shaped windows rotated at variable angles; (7) circular windows at variable centre positions; (8) sinusoidal gratings with variable periods and orientations; (9) Fresnel zone plates with variable x-y spatial positions; and (10) superpositions of Gaussian functions and square windows at variable spatial x-y positions.

For the second type of D^2^NN with a Fourier plane input filter, using the same Rayleigh-Sommerfeld diffraction equation mentioned above, we numerically implemented a 4-f system with two lenses; the first lens transforms the object information from the spatial domain to the frequency domain, and the second lens does the opposite. On the Fourier plane that is 2f distance away from the object plane, a single amplitude-only input filter, designed in one of the following forms, is employed: (1) various combinations of circular/annular passbands, which are defined by specifying a series of equally spaced concentric ring-like areas, such that it can serve as a low/high-pass, single-band-pass or multi-band-pass filter or (2) a single trainable layer enabling the system to learn an input spatial frequency filter on its own. On the output image plane of the 4-f system that is 4f distance away from the object plane, a square aperture is placed with the same size as the object or 1.5 times the size of the object before feeding the resulting complex-valued field into the diffractive network. In the numerical implementation, the lens has a focal length *f* of ~145.6 λ and a diameter of 104 λ.

For each type of input filter design, the number of trained base D^2^NNs and some input filter examples can be found in Supplementary Table S1.

### Training details

All the D^2^NNs and their weighted ensembles in this paper were numerically implemented and trained using Python (v3.6.5) and TensorFlow (v1.15.0, Google). An Adam optimizer^50^ with the default parameters from TensorFlow was used to calculate the back-propagated gradients during the training of the individual optical models and the ensemble weights. The learning rate, starting from an initial value of 0.001, was set to decay at a rate of 0.7 every 8 epochs. The publicly available CIFAR-10 dataset consists of 50,000 training images and 10,000 test images^35^. The training images were split into sets of 45,000 and 5000 images for training and validation, respectively. All the blind testing accuracies reported in this paper (individual D^2^NN and ensemble models) were evaluated on the 10,000 test images, which were never used during the training of the individual networks nor during the optimization of the weights for the ensemble pruning (detailed in the following subsection). Since the images in the original CIFAR-10 dataset contain three colour channels (red, green and blue) and monochromatic illumination is used in our diffractive optical network models, the built-in *rgb_to_grayscale* function in TensorFlow was applied to convert these colour images to greyscale. In addition, to enhance the generalization capability of the trained D^2^NNs, we randomly flipped the images (left to right) with a probability of 0.5 while training. For training the individual D^2^NNs, we used a batch size of 8, trained each model for 50 epochs using the training image set and selected the best model based on the classification performance on the validation image set. The D^2^NN loss function for a given network *k* was the softmax-cross-entropy between the differential class scores *z*_*ck*_ and their one-hot labelled ground-truth vector *g*:

2 $${\mathrm{D}}^{\mathrm{2}}{\mathrm{NN}}\;{\mathrm{Loss}} = - E\left[ {\mathop {\sum}\limits_{c = 1}^C {g_c\log \left( {\frac{{\exp \left( {z_{ck}} \right)}}{{\mathop {\sum}\nolimits_{c = 1}^C {\exp \left( {z_{ck}} \right)} }}} \right)} } \right]$$

where *E*[.] denotes the expectation over the training images in the current batch, *C*=10 denotes the total number of classes in the dataset, and *g*_*c*_ represents the *c*^th^ entry of the ground-truth label vector *g*.

### Ensemble pruning

The method we followed for ensemble pruning involved iterative elimination of the D^2^NN members from the initial pool of 1252 unique networks based on a quantitative metric, which is indicative of an individual network’s ‘significance’ in the collective inference process. However, since a member’s individual performance supremacy might not always translate to an improvement in the ensemble, during the iterative process, we occasionally eliminated some members randomly. Ensemble pruning with intermittent random elimination of members was found to result in better performing ensembles compared to pruning without random elimination, as detailed in the “Results” section and Table 1.

**Fig. 4:**
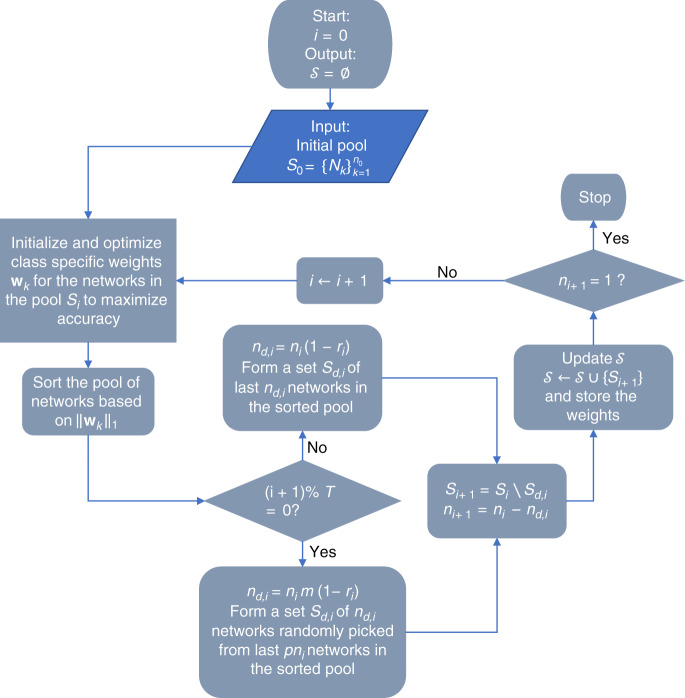
Flow chart of the ensemble pruning process. The meaning of the symbols is as follows: *i* is the iteration number; *S* is the set of ensembles, resulting after each iteration; *S*_*i*_ is the ensemble after iteration *i*; *n*_*i*_ is the number of networks in the ensemble after iteration *i*; *w*_*k*_ is the weight vector for networ*k k*; *T* is the interval between the random eliminations of D^2^NNs; *S*_*d,i*_ is the set of networks to eliminate from the ensemble in iteration *i*; *n*_*d,i*_ is the number of networks to eliminate from the ensemble in iteration *i*; *r*_*i*_ is the fraction of networks to retain in iteration *i*; *m* is the ratio of the number of randomly eliminated networks to the number of networks eliminated based on ranking; *p* is the fraction of the networks in the ensemble to which random elimination is applied. At the end of the pruning process, *S* comprises a series of D^2^NN ensembles (formed by *S*_*i*_) of gradually decreasing size

Our pruning method (see Fig. 4) was initiated with an ensemble that consisted of all the *n*_0_ = 1252 individually trained D^2^NN models. An ensemble class score *z*_*c*_ was defined as:

3 $$z_c = {\sum \limits_{\mathrm{k}}} {w_{ck}z_{ck}}$$

where *z*_*ck*_ is the score predicted for class *c* by member/network *k* (Eq. 1) and *w*_*ck*_ is the corresponding class-specific weight. The weight vectors $w_k = \left\{ {w_{ck}} \right\}_{c = 1}^C$, *k* = 1, 2, …, *n*_0_, were optimized by minimizing the softmax-cross-entropy loss of the class scores predicted by the ensemble of D^2^NNs; *C*=10 denotes the total number of classes in our dataset. To reduce overfitting of the weights to the training data examples, an L2 loss term was also included in our pruning loss function:

4 $${\mathrm{Pruning}}\;{\mathrm{loss}} = - E\left[ {\mathop {\sum}\limits_{c = 1}^C {g_c\log \left( {\frac{{\exp \left( {z_c} \right)}}{{\mathop {\sum}\nolimits_{c = 1}^C {\exp \left( {z_c} \right)} }}} \right)} } \right] + \alpha \left( {\frac{1}{2}\mathop {\sum}\limits_{k = 1}^{n_0} {\mathop {\sum}\limits_{c = 1}^C {w_{ck}^2} } } \right)$$

where *α* is set to 0.001, *E*[.] denotes the expectation over the image batch, and *g*_*c*_ represents the *c*^th^ entry of the ground-truth label vector *g*. During the optimization of the ensemble, in each iteration of the backpropagation algorithm, all the image samples in the validation set were fed into the ensemble model (i.e., the batch size equals 5 K); using training images for weight optimization during ensemble pruning resulted in overfitting and therefore was not implemented. The class-specific weights were optimized using the gradient descent algorithm (Adam^50^) for 10,000 steps. After optimizing the weights, the individual members/networks were ranked based on a quantitative metric. An intuitive choice for this metric is the individual prediction accuracy of each network. However, a better metric for measuring the significance of individual networks in an ensemble was found to be the L1 norm of the individual weight vectors optimized for the validation accuracy. The superiority of the weight L1 norm as a metric was substantiated by the fact that it consistently resulted in ensembles achieving much better blind testing accuracies. After ranking the members based on their weight vectors, a certain fraction of them was eliminated from the bottom (i.e., the lowest-ranked members), and the procedure was repeated with the reduced ensemble until only one member was left in the ensemble. As mentioned earlier, at every *T*-th iteration of the pruning process, this member/network elimination was performed *randomly* instead of via ranking-based elimination. However, to avoid elimination of the members with the largest weights, random elimination was selected within a fraction *p* of the networks counted from the bottom; *p* was 2/3 in our case. Once the pruning process was complete (see Fig. 4), a maximum allowable ensemble size (*N*_max_) was set, and the ensemble with the best performance on the validation dataset and satisfying the size limit was chosen. The test image dataset was never used during the pruning process.

To further explore an extended weight assignment scheme, we used a modified version of Eq. 3:

5 $$z_c = \mathop {\sum}\nolimits_{\mathrm{k}} {w_{ck}\frac{{w_{ck}^ + z_{ck}^ + - w_{ck}^ - z_{ck}^ - }}{{w_{ck}^ + z_{ck}^ + + w_{ck}^ - z_{ck}^ - }}}$$

where $w_{ck}^ +$ and $w_{ck}^ -$ are the newly introduced weights assigned to the positive and the negative detector of each detector pair, respectively. Accordingly, the pruning loss defined in Eq. 4 was changed to be:

6 $${\mathrm{Pruning}}\;{\mathrm{loss}} = - E\left[ {\mathop {\sum}\limits_{c = 1}^C {g_c\log \left( {\frac{{\exp \left( {z_c} \right)}}{{\mathop {\sum}\nolimits_{c = 1}^C {\exp \left( {z_c} \right)} }}} \right)} } \right] + \alpha \left( {\frac{1}{2}\mathop {\sum}\limits_{k = 1}^{n_0} {\mathop {\sum}\limits_{c = 1}^C {w_{ck}^2} } } \right) + \beta \left( {\frac{1}{2}\mathop {\sum}\limits_{k = 1}^{n_0} {\mathop {\sum}\limits_{c = 1}^C {w_{ck}^{ - 2}{\mathrm{ + }}w_{ck}^{ + 2}} } } \right)$$

where and *β* are both empirically set to 0.001. During the pruning process, when weight assignment scheme (1) described in the Results section was used, all the weights *w*_*ck*_, $w_{ck}^ +$ and $w_{ck}^ -$ were simultaneously optimized for 10,000 iterations. In weight assignment scheme (2) described in the “Results” section, the optimization of *w*_*ck*_ and ($w_{ck}^ +$, $w_{ck}^ -$) was performed alternatively; each time, one group of weights was optimized for 100 iterations, and in total, 50 cycles were used to obtain an equivalent number of total iterations (10,000), the same as in scheme (1).

For all the training and optimization tasks detailed above, we used multiple desktop computers all with one or two GTX 1080 Ti graphical processing units (GPUs, Nvidia Inc.), Intel® Core™ i7-8700 central processing units (CPUs, Intel Inc.) and 64 GB of RAM, running the Windows 10 operating system (Microsoft Inc.). The typical training time for one D^2^NN model on a single GPU is ~3 h. The time required for the iterative ensemble pruning process depends on the pruning hypermeters, varying between 0.75 and 7.5 h.

## Supplementary information

Supplementary Information

## Data Availability

All the data and methods needed to evaluate the conclusions of this work are presented in the main text and the Supplementary Information. Additional data can be requested from the corresponding author.

## Code availability

The deep learning models reported in this work used standard libraries and scripts that are publicly available in TensorFlow.
