# Publisher Correction: Ensemble learning of diffractive optical networks

**DOI:** 10.1038/s41377-021-00473-1

**Published:** 2021-02-07

**Authors:** Md Sadman Sakib Rahman, Jingxi Li, Deniz Mengu, Yair Rivenson, Aydogan Ozcan

**Affiliations:** 1 Electrical and Computer Engineering Department, University of California, Los Angeles, CA 90095 USA; 2 Bioengineering Department, University of California, Los Angeles, CA 90095 USA; 3 California NanoSystems Institute (CNSI), University of California, Los Angeles, CA 90095 USA

**Keywords:** Imaging and sensing, Applied optics, Optical physics

Correction to: *Light: Science & Applications*

10.1038/s41377-020-00446-w published online 11 Jan 2021

After publication of this article^1^, it is noticed that the article contained some typographical errors:

1. In Table 1 caption text, “D2NN” should have been “D^2^NN,” where 2 is superscript.
2. In the “Materials and Methods” section, “where and *β* are…” should read as “where *α* and *β* are…”.

These typographical errors were introduced during the editorial production and were not part of the authors’ original manuscript.
