# Supplementary material for: Ensemble learning of diffractive optical networks
Source: Light Sci Appl. 2021 Jan 11;10:14. doi: 10.1038/s41377-020-00446-w (PMC7801728; doi:10.1038/s41377-020-00446-w)
Supplement: Supplementary file 1 — Supplementary Information [file 41377_2020_446_MOESM1_ESM.pdf]

Supplementary Information for

Ensemble learning of diffractive optical networks

Md. Sadman Sakib Rahman[1,2,3,†] email: mssr@ucla.edu

Jingxi Li[1,2,3,†] email: jxlli@ucla.edu

Deniz Mengu[1,2,3] email: denizmengu@ucla.edu

Yair Rivenson[1,2,3] email: rivensonyair@ucla.edu

Aydogan Ozcan[1,2,3,*] email: ozcan@ucla.edu

telephone: +1 310-825-0915

[1]Electrical and Computer Engineering Department, University of California, Los Angeles, CA, 90095, USA

[2]Bioengineering Department, University of California, Los Angeles, CA, 90095, USA

[3]California NanoSystems Institute (CNSI), University of California, Los Angeles, CA, 90095, USA

[†]Equal contributing authors

[*]Corresponding author: ozcan@ucla.edu

Supplementary Figures

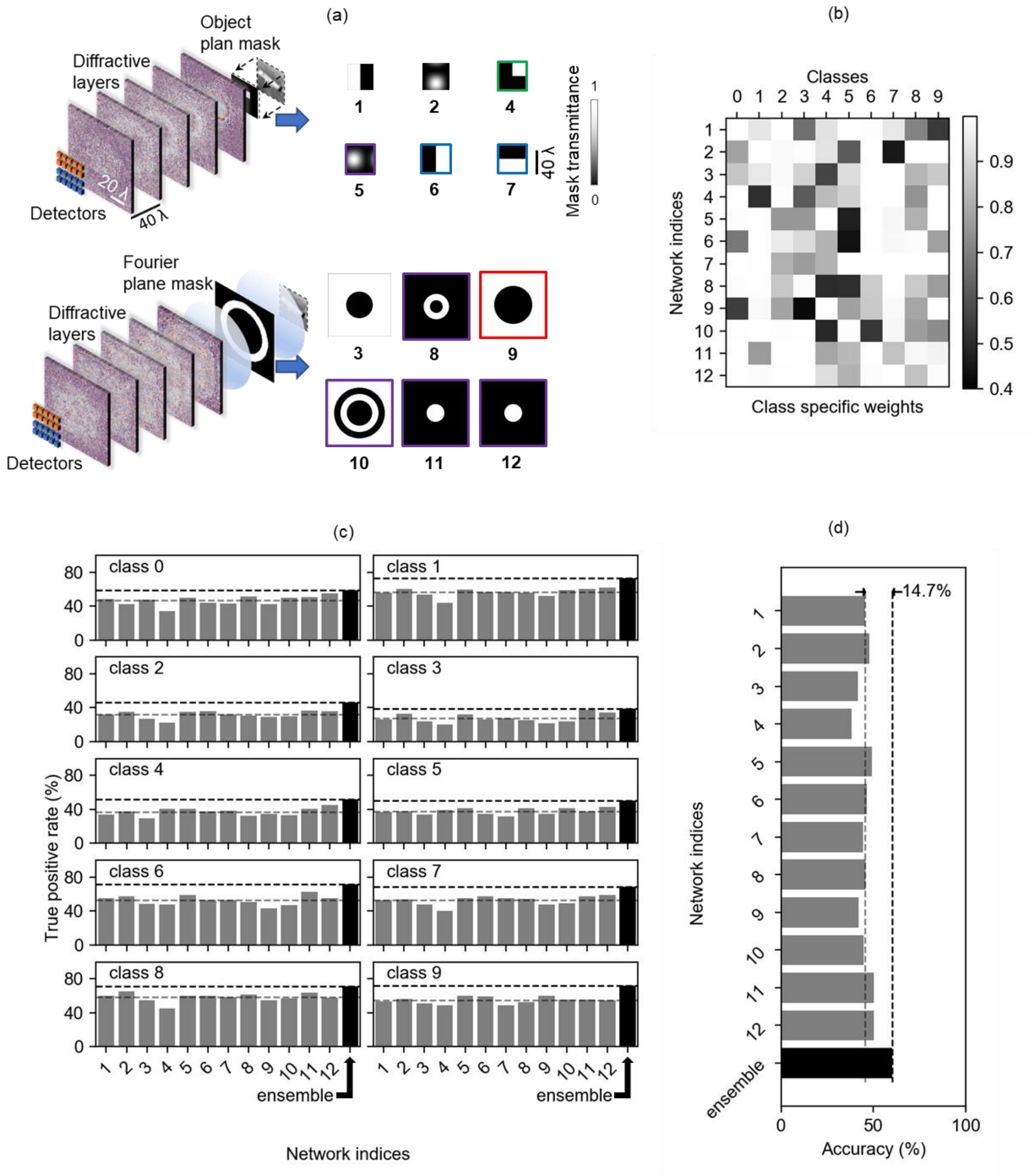

Fig. S1 An ensemble of N=12 D²NNs achieves a blind classification accuracy of 60.29% on CIFAR-10 test dataset. (a) Input filters/masks used before each one of the D²NNs that form the ensemble. For D²NNs 1, 2, 4-7: the input filters are on the object plane. For the remaining D²NNs 3, 8-12: the input filters are on the Fourier plane. The input filters corresponding to the networks

with phase encoded input are enclosed within a border/frame (4-12), while the inputs of the diffractive networks 1-3 are amplitude encoded. The dynamic range of the input phase encoding is represented by the border color; red: 0-π/2, green: 0-π, blue: 0-3π/2, purple: 0-2π. (b) Class specific weights for each D^2NN of the ensemble. (c) True positive rates of the individual networks, compared against their ensemble for different classes. (d) Test accuracy of the individual networks compared against their ensemble. The dotted lines show the classification performance improvement (~14.7%) achieved by the diffractive ensemble over the mean performance of the individual D^2NNs. Three repeats with the same hyperparameters resulted in a blind classification accuracy of 60.35±0.39%, where 60.29% represents the median.

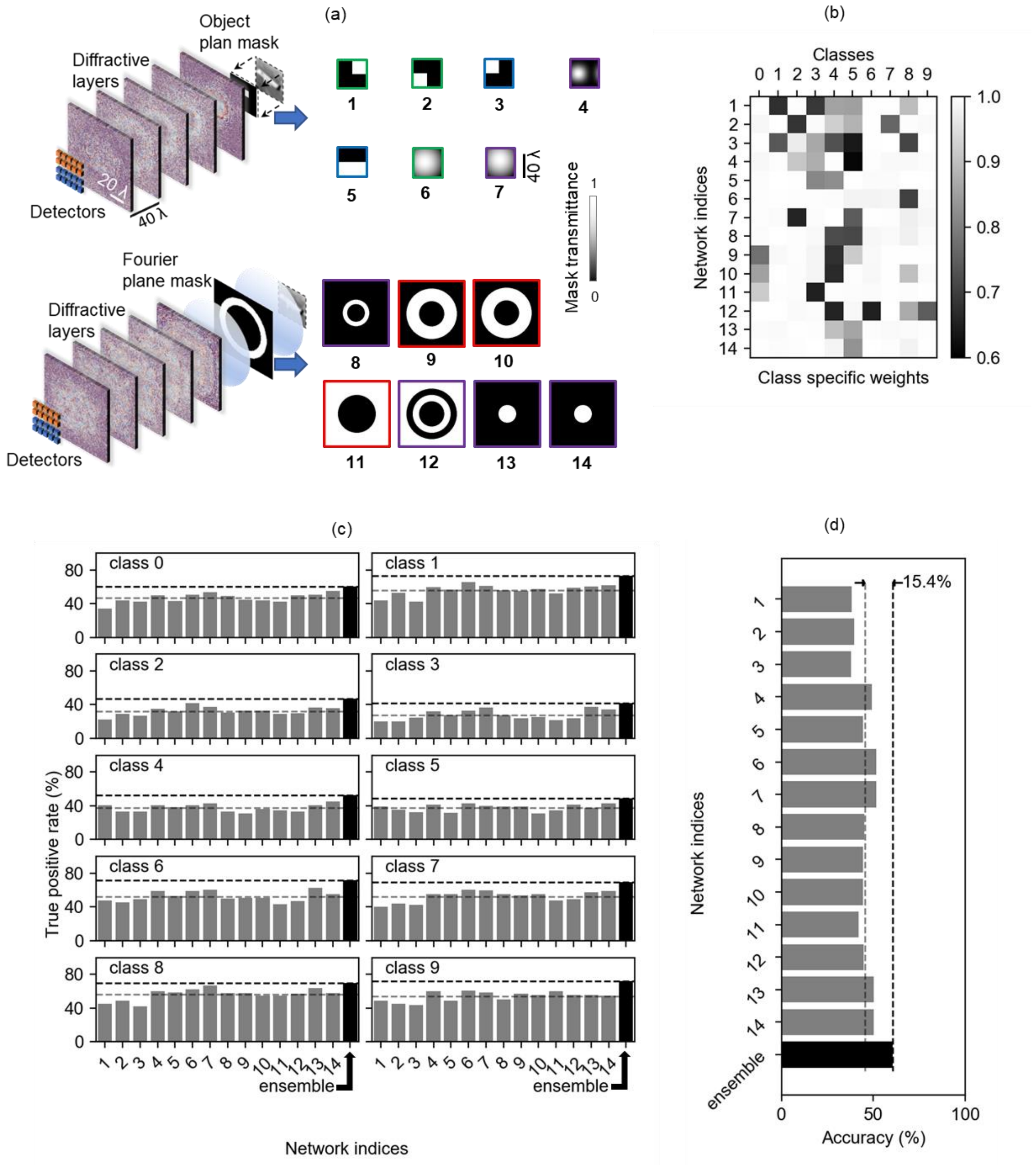

Fig. S2 A D²NN ensemble consisting of only phase-encoded-input networks (N=14) achieves a blind classification accuracy of 60.65% on CIFAR-10 test dataset. (a) Input filters/masks used before each one of the D²NNs that form the ensemble. For D²NNs1-7: the input filters are on the object plane. For the remaining D²NNs 8-14: the input filters are on the Fourier plane. The dynamic range of the input phase encoding is represented by the border color; red: 0-π/2, green: 0-π, blue: 0-3π/2, purple: 0-2π. (b) Class specific weights for each D²NN of the ensemble. (c) True

positive rates of the individual networks, compared against their ensemble for different classes. (d) Test accuracy of the individual networks compared against their ensemble. The dotted lines show the classification performance improvement (~15.4%) achieved by the diffractive ensemble over the mean performance of the individual D^2NNs. Three repeats with the same hyperparameters resulted in a blind classification accuracy of 60.74±0.17%, where 60.65% represents the median.

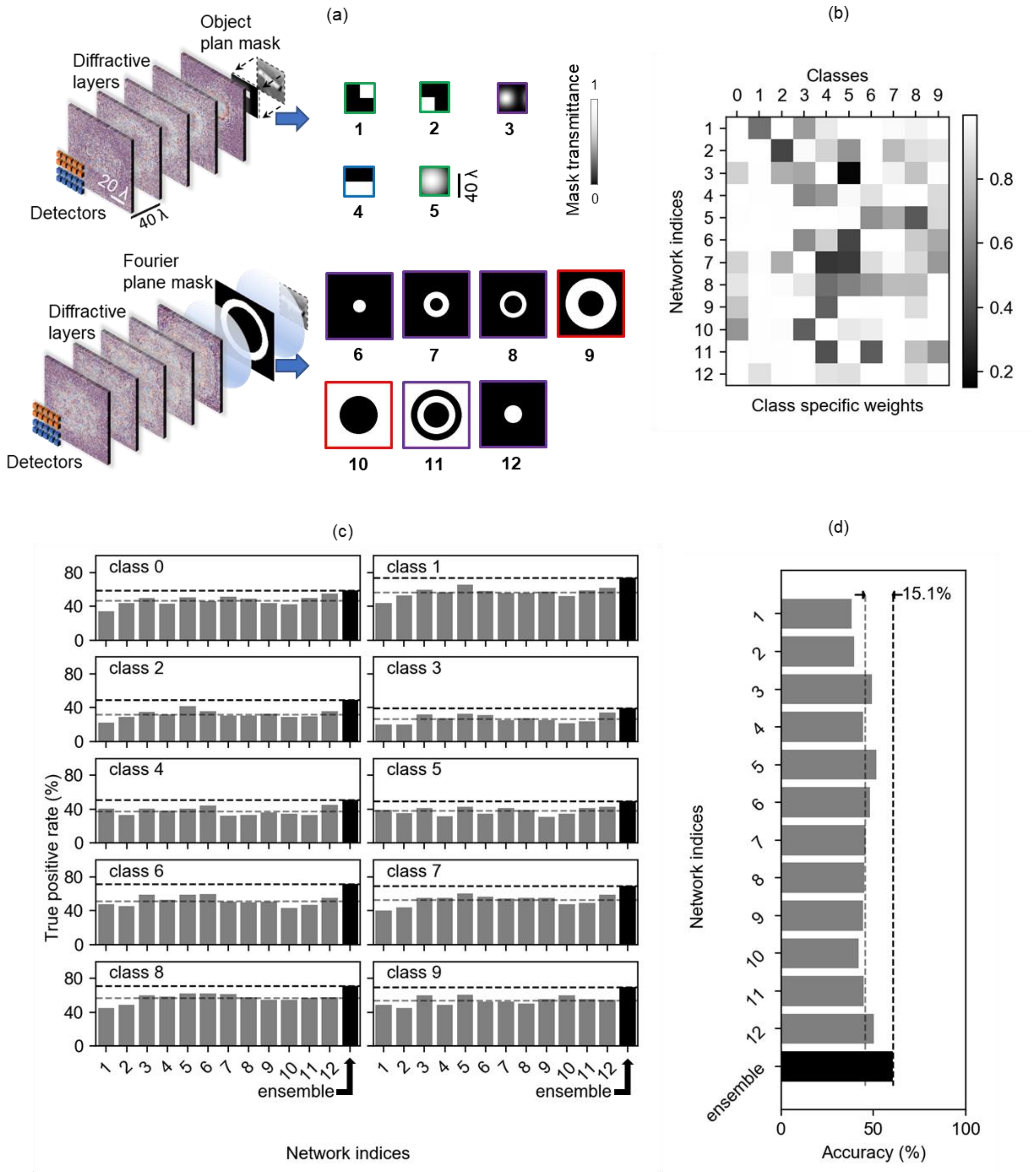

Fig. S3 A D²NN ensemble consisting of only phase-encoded-input networks (N=12) achieves a blind classification accuracy of 60.43% on CIFAR-10 test dataset. (a) Input filters/masks used before each one of the constituent D²NNs that form the ensemble. For D²NNs 1-5: the input filters are on the object plane. For the remaining D²NNs 6-12: the input filters are on the Fourier plane. The dynamic range of the input phase encoding is represented by the border/frame color; red: 0-π/2, green: 0-π, blue: 0-3π/2, purple: 0-2π. (b) Class specific weights for each D²NN of the

ensemble. (c) True positive rates of the individual networks, compared against their ensemble for different classes. (d) Test accuracy of the individual networks compared against their ensemble. The dotted lines show the classification performance improvement (~15.1%) achieved by the diffractive ensemble over the mean performance of the individual D^2NNs. Three repeats with the same hyperparameters resulted in a blind classification accuracy of 60.41±0.10%, where 60.43% represents the median.

Supplementary Tables

Type	Index	Description	Number of trained base D²NNs	Example of masks
Masks placed on the object plane	1)	2D Gaussian function defined with variable shapes and spatial x-y positions	310	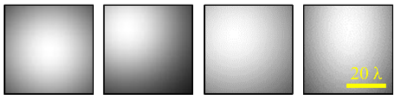
	2)	Multiple superposed 2D Gaussian function defined with variable spatial x-y positions	50	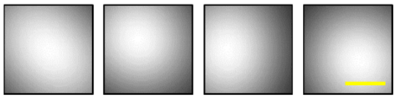
	3)	2D Hamming/Hanning function defined with variable spatial x-y positions	50	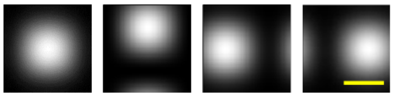
	4)	Square windows with different sizes at variable spatial x-y positions	234	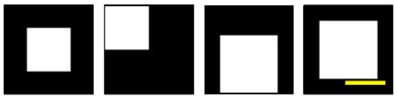
	5)	Multiple square windows at variable spatial x-y positions	50	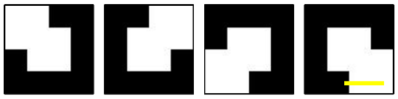
	6)	Patch shaped windows rotated at variable orientations	40	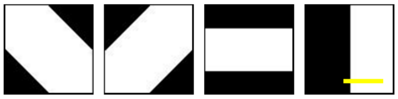
	7)	Circular windows at variable spatial x-y positions	45	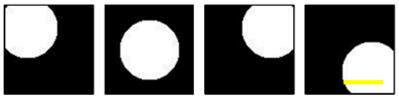
	8)	Sinusoidal gratings with variable periods and orientations	48	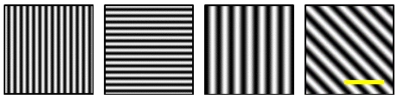
	9)	Fresnel zone plates with variable x-y spatial positions	45	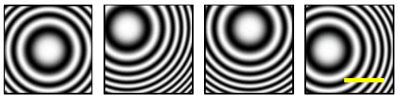
	10)	Superposition of Gaussian functions and square windows at variable spatial x-y positions	60	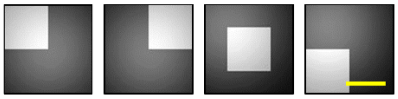
Masks placed on the Fourier plane	11)	Combination of circular/annular transmissive/non-transmissive zones serving as a low pass, high pass or multi-band pass filter	300	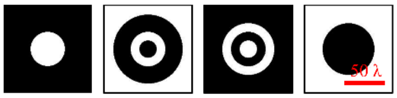
	12)	A single trainable transmissive layer	20	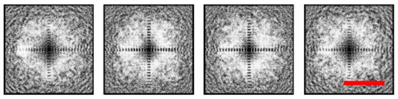
			Total = 1252	Mask transmittance 0 – 1

Table S1 The initial network pool containing 1252 individually-trained D²NNs. For each type of the input mask design, a brief description is given, and the number of trained base D²NN classifiers and some examples are presented.

Accuracy (%) / Number of networks / Accuracy per network (%)	T	10			20			∞		
	r_i	(i)	(ii)	(iii)	(i)	(ii)	(iii)	(i)	(ii)	(iii)
m	1	60.437 ±0.067	60.433 ±0.045	60.513 ±0.121	60.740 ±0.165	60.560 ±0.182	60.410 ±0.177	60.360	60.360	60.570
		14	14	14	14	14	14	14	14	14
		4.317 ±0.005	4.317 ±0.003	4.322 ±0.009	4.339 ±0.012	4.326 ±0.013	4.315 ±0.013	4.311	4.311	4.326
	3	60.700 ±0.149	60.667 ±0.163	60.630 ±0.328	60.430 ±0.286	60.627 ±0.410	60.663 ±0.076	60.360	60.360	60.570
		14	14	13	13	14	14	14	14	14
		4.336 ±0.011	4.333 ±0.012	4.664 ±0.025	4.648 ±0.022	4.330 ±0.029	4.333 ±0.005	4.311	4.311	4.326
	10	60.413 ±0.096	60.700 ±0.159		60.620 ±0.046	60.553 ±0.163		60.360	60.360	60.570
		12	14		14	14		14	14	14
		5.034 ±0.008	4.336 ±0.011		4.330 ±0.003	4.325 ±0.012		4.311	4.311	4.326

Table S2 Comparison of blind testing accuracy results achieved under different pruning hyperparameters, with only phase encoded input D^2NNs and a maximum allowed ensemble size of N_{max}=14 (see Fig. 4 of main text). For the classification accuracies that are reported, the average and the standard deviation values result from 3 independent repeats of the pruning process using the same hyperparameters. The green box highlights the D^2NN ensemble achieving the best average blind testing accuracy (N=14), and the red box highlights the D^2NN ensemble achieving the best average blind testing accuracy *per network* (N=12).

Accuracy (%) / Number of networks / Accuracy per network (%)	T	10			20			∞		
	r_i	(i)	(ii)	(iii)	(i)	(ii)	(iii)	(i)	(ii)	(iii)
m	1	60.520 ±0.135	60.757 ±0.118	60.770 ±0.175	60.780 ±0.105	60.877 ±0.232	60.967 ±0.061	60.770	60.850	60.770
		14	14	14	14	14	14	14	14	14
		4.323 ±0.010	4.340 ±0.008	4.341 ±0.013	4.341 ±0.008	4.348 ±0.017	4.355 ±0.004	4.341	4.346	4.341
	3	61.007 ±0.182	60.617 ±0.075	60.833 ±0.201	60.933 ±0.224	60.510 ±0.101	60.913 ±0.108	60.770	60.850	60.770
		14	14	14	14	12	14	14	14	14
		4.358 ±0.013	4.330 ±0.005	4.345 ±0.014	4.352 ±0.016	5.042 ±0.008	4.351 ±0.008	4.341	4.346	4.341
	10	60.927 ±0.206	60.820 ±0.242		61.023 ±0.289	60.607 ±0.222		60.770	60.850	60.770
		14	14		14	12		14	14	14
		4.352 ±0.015	4.344 ±0.017		4.359 ±0.021	5.051 ±0.019		4.341	4.346	4.341

Table S3 Comparison of blind testing accuracy results achieved under different pruning hyperparameters, with a maximum allowed ensemble size of N_{max}=14, where in the pruning process trainable weights are also assigned to the individual detector signals and are *simultaneously* optimized with the class score weights (referred to as the weight assignment scheme (1) in the Results section). For the classification accuracies that are reported, the average and the standard deviation values result from 3 independent repeats of the pruning process using the same hyperparameters. The green box highlights the D^2NN ensemble achieving the best average blind testing accuracy (N=14), and the red box highlights the D^2NN ensemble achieving the best average blind testing accuracy *per network* (N=12).

Accuracy (%) / Number of networks / Accuracy per network (%)	T	10			20			∞		
	r_i	(i)	(ii)	(iii)	(i)	(ii)	(iii)	(i)	(ii)	(iii)
m	1	60.920 ±0.166	60.913 ±0.179	60.953 ±0.289	60.937 ±0.131	61.103 ±0.263	60.883 ±0.091	60.610	61.270	61.350
		14	14	14	14	14	14	13	14	14
		4.351 ±0.012	4.351 ±0.013	4.354 ±0.021	4.353 ±0.009	4.365 ±0.019	4.349 ±0.006	4.662	4.376	4.382
	3	60.710 ±0.529	60.793 ±0.215	60.973 ±0.085	61.010 ±0.195	60.923 ±0.158	61.033 ±0.137	60.610	61.270	61.350
		14	14	14	14	14	14	13	14	14
		4.336 ±0.038	4.342 ±0.015	4.355 ±0.006	4.358 ±0.014	4.352 ±0.011	4.360 ±0.010	4.662	4.376	4.382
	10	60.767 ±0.121	60.940 ±0.193		61.227 ±0.244	60.177 ±0.734		60.610	61.270	61.350
		14	14		14	12		13	14	14
		4.340 ±0.009	4.353 ±0.014		4.373 ±0.017	5.015 ±0.061		4.662	4.376	4.382

Table S4 Comparison of blind testing accuracy results achieved under different pruning hyperparameters, with a maximum allowed ensemble size of N_{max}=14, where in the pruning process trainable weights are also assigned to the detector signals and are *alternatively* optimized with the class score weights (referred to as the weight assignment scheme (2) in the Results section. For the classification accuracies that are reported, the average and the standard deviation values result from 3 independent repeats of the pruning process using the same hyperparameters. The green box highlights the D^2NN ensemble achieving the best average blind testing accuracy (N=14), and the red box highlights the D^2NN ensemble achieving the best average blind testing accuracy *per network* (N=12).
